# SMaSH: Sample matching using SNPs in humans

**DOI:** 10.1186/s12864-019-6332-7

**Published:** 2019-12-30

**Authors:** Maximillian Westphal, David Frankhouser, Carmine Sonzone, Peter G. Shields, Pearlly Yan, Ralf Bundschuh

**Affiliations:** 1 Interdisciplinary Biophysics Graduate Program, The Ohio State University, 484 W. 12th Avenue, Columbus, 43210 OH USA; 2 Biomedical Science Graduate Program, The Ohio State University, 333 W. 10th Avenue, Columbus, 43210 OH USA; 3 Department of Diabetes Complications and Metabolism and Department of Population Sciences in the Beckman Research Institute, City of Hope, 1500 East Duarte Road, Duarte, 91010 CA USA; 4 Molecular, Cellular, and Developmental Biology Graduate Program, The Ohio State University, 484 W. 12th Avenue, Columbus, 43210 OH USA; 5 Department of Internal Medicine, The Ohio State University, 395 W. 12th Avenue, Columbus, 43210 OH USA; 6 Comprehensive Cancer Center, The Ohio State University, 460 W. 10th Avenue, Columbus, 43210 OH USA; 7 Department of Physics, The Ohio State University, 191 W. Woodruff Avenue, Columbus, 43210 OH USA; 8 Department of Chemistry and Biochemistry, The Ohio State University, 100 W. 18th Avenue, Columbus, 43210 OH USA; 9 Center for RNA Biology, The Ohio State University, 484 W. 12th Avenue, Columbus, 43210 OH USA

**Keywords:** Sample swap, Next generation sequencing data, Identity matching

## Abstract

**Background:**

Inadvertent sample swaps are a real threat to data quality in any medium to large scale omics studies. While matches between samples from the same individual can in principle be identified from a few well characterized single nucleotide polymorphisms (SNPs), omics data types often only provide low to moderate coverage, thus requiring integration of evidence from a large number of SNPs to determine if two samples derive from the same individual or not.

**Methods:**

We select about six thousand SNPs in the human genome and develop a Bayesian framework that is able to robustly identify sample matches between next generation sequencing data sets.

**Results:**

We validate our approach on a variety of data sets. Most importantly, we show that our approach can establish identity between different omics data types such as Exome, RNA-Seq, and MethylCap-Seq. We demonstrate how identity detection degrades with sample quality and read coverage, but show that twenty million reads of a fairly low quality RNA-Seq sample are still sufficient for reliable sample identification.

**Conclusion:**

Our tool, SMASH, is able to identify sample mismatches in next generation sequencing data sets between different sequencing modalities and for low quality sequencing data.

## Background

Because no laboratory tracking method is perfect, there is always a risk of error in sample identification in next generation sequencing (NGS), which increases as the size and scope of a study increases [1]. Sequencing Core Laboratories and Genomic Centers utilize different instruments and protocols from center to center [2]. E.g., upwards of seven different protocols exist for RNA-Seq alone and there are even variations in how these protocols are executed [3]. As the number of steps in a protocol increase, and large numbers of samples are processed together, so does the chance for sample mix-up, sample cross-contamination or the wrong barcode-adapter being assigned to a sample for indexing. Tools such as MODMatcher [4] or MixupMapper [5] have found several TCGA and LGRC microarray data sets that have sample misidentification rates as high as 6.5%.

While there are methods for ensuring that a NGS sample on the flow cell is the same sample that arrived at the sequencing center, there is no method for validating whether a sample that has already been sequenced is the correct sample. Also, inadvertent sample swaps can already occur before delivering samples to the sequencing facility. In tumor-normal, knock down/knock out analysis in primary cultures, or drug trial studies, an incorrectly identified sample can have egregious effects on the resulting data.

Existing sample identification methods, such as STR profiling [6] or SNP panels [7, 8], require additional data and experiments to validate whether a sample is correctly identified. While a list of 20-45 SNPs [9, 10] could be used to identify a sample, it is too narrow of a list to reliably use in RNA-Seq experiments, which have varying read coverage based on gene expression and thus often do not allow reliable genotyping of a given SNP. Thus, with currently available methods and pipelines, an RNA-Seq experimenter might be forced to pay for more experiments in order to verify their samples’ identity.

Here we present SMaSH (Sample Matching using SNPs in Humans). Unlike existing methods, such as STR profiling, SNP panels, or post hoc computational analysis of microarray data, SMaSH uses human-aligned NGS data to determine whether or not two or more samples were derived from the same patient. SMaSH uses a similar statistical approach as presented by Korneliussen et al. [11] for the purpose of estimating kinship from NGS data. However, in contrast to this previous work, SMaSH is specifically geared to patient sample identity detection rather than general kinship determination. It utilizes a carefully selected set of SNPs from across the genome to ensure that enough data points exist to call sample pairings while keeping the total number of SNPs at a level that would be amenable to storing the information extracted at these SNPs as a “fingerprint” of the sample in a database. Most importantly, we show that SMaSH is able to compare *across* data types and has been able to successfully identify matches between RNA-seq data, Exome data, and MethylCap-seq data that were derived from the same patient. This latter ability is crucial for quality assurance in modern multimodal omics studies.

## Results

### Approach

In order to computationally identify samples that are derived from the same individual, we select a set of common SNPs that we use as the genetic fingerprint of the individual. In order to maximize our ability to apply our method to data sets from different library preparation methods we select SNPs in genomic locations that are covered by exome sequencing and various RNA sequencing approaches. We also enforce a minimal distance between neighboring SNPs to minimize effects of linkage disequilibrium. These principles yielded a set of 6059 SNPs (see “Methods” section for details).

**Fig. 1:**
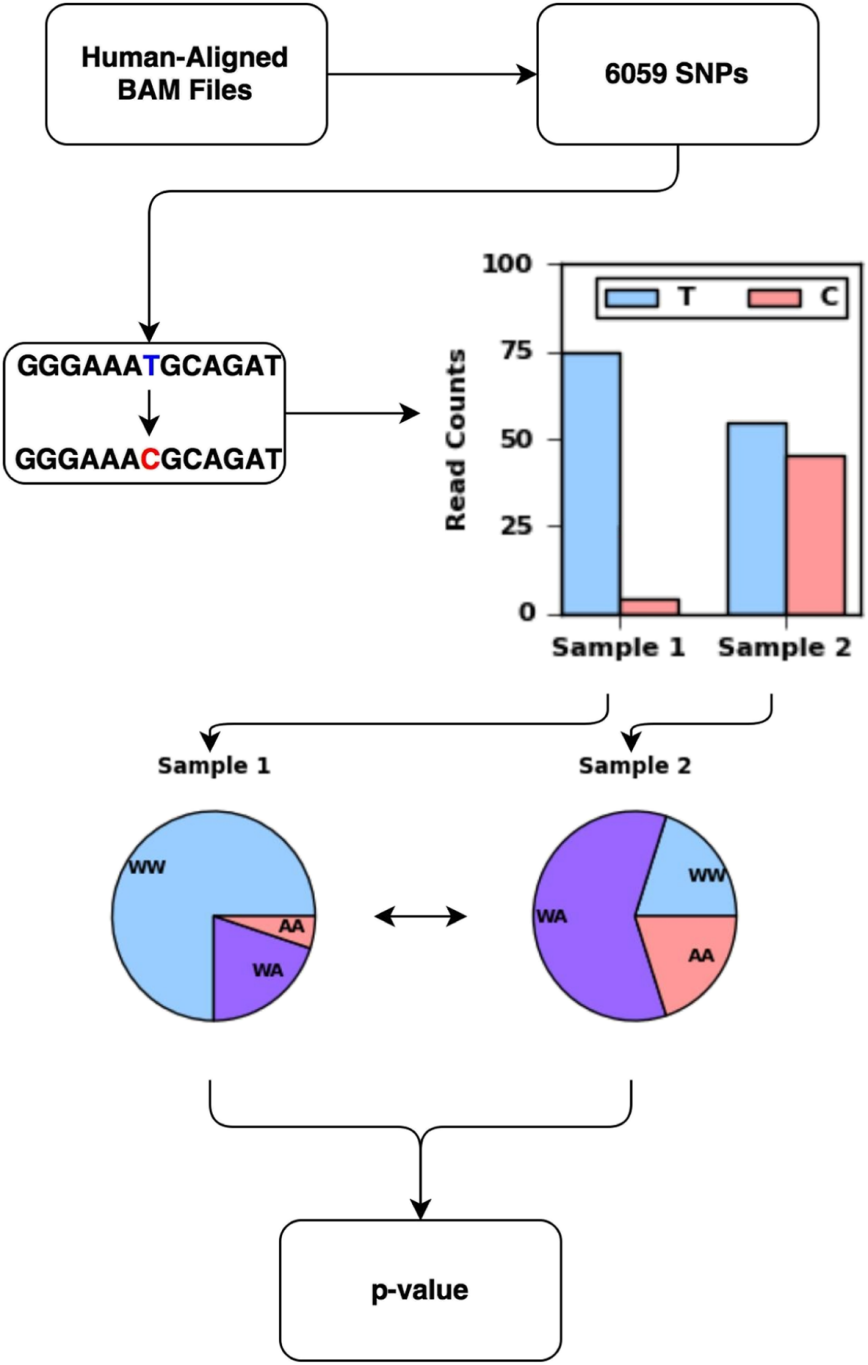
Workflow of SMaSH. The number of reads supporting the wild type and alternate allele at 6059 SNPs in the human genome are counted and a Bayesian approach is used to calculate a *p*-value for the null hypothesis that the two samples are derived from the same individual. W = Wild Type; A = Alternate Allele

Figure 1 then shows how two samples are compared with each other. We first count the reads supporting the reference and non-reference alleles at each of the selected SNP locations, realizing that for a possibly large number of these locations in any given comparison these counts may be zero or very low. We then use a Bayesian approach to calculate the probability that the read counts were generated from samples from the same individual (see “Methods” section for details). This Bayesian approach aggregates the evidence from all SNP locations automatically giving more weight to locations with high read counts than locations with low read counts. The final result of this calculation is a *p*-value for the null hypothesis that the two samples are derived from the same individual. If this *p*-value is below a preestablished threshold, our tool SMaSH identifies the two samples as coming from different individuals.

We provide an implementation of SMaSH in python 2.7 on github at http://github.com/rbundschuh/SMaSH. The README file displayed at that url also provides explicit instructions on how to run our software.

Since SMaSH is a classifier, its performance is best quantified in terms of the Receiver Operator Characteristic (see “Methods” section). In order to demonstrate that SMaSH is indeed able to identify sample matches in different sequencing data types, its performance was assessed on several in house and public data sets of varying quality and data type. We discuss the relevant results for each data set below; Additional file 1 contains the individual *p*-values for each comparison.

### Performance on high quality datasets of different sequencing approaches

We started by evaluating SMaSH on three high quality data sets, which are described in some more detail below. For each of these data sets SMaSH provided perfect classification, i.e., all pairings involving samples from the same individuals had higher *p*-values than all pairings involving samples from different individuals. We thus do not show the respective ROC plots. The first of these three data sets consisted of 24 whole transcriptome RNA-Seq samples evenly distributed among 12 patients and is available under SRA accession SRP076801. Each patient had one pair of tumor-normal samples and no other associated samples. The *p*-values for all sample pairings involving identical patients were numerically indistinguishable from one; the largest *p*-value for a sample pair involving different patients was below 10^−102^ (i.e., the *p*-values for all sample pairs involving different patients were between 0 and 10^−102^). The second and third data set tested were meant to evaluate the ability of SMaSH to identify patient identity between different sequencing data types. The second data set consisted of 149 samples distributed among 23 patients. Each patient had both MethylCap-seq and RNA-Seq samples sequenced at up to 5 timepoints. These two different data types require very different downstream analysis and library generation protocols, yet SMaSH was able to perfectly identify samples from the same patients under these circumstances. The lowest *p*-value for a sample pair from the same individual was 0.999999997 (i.e., all *p*-values of sample pairs from the same individual were between 0.999999997 and 1) and the highest *p*-value for a sample pair from different individuals was 0.50 indicating that this data set was more difficult to classify than the first one. The third data set was obtained from TCGA and consisted of 20 lung squamous cell carcinoma samples evenly distributed among 10 patients. Each patient had one RNA-seq sample generated at the UNC-LCCC and one Exome sample generated at the Broad Institute. Here, again the *p*-values for all sample pairings involving identical patients were numerically indistinguishable from one; the largest *p*-value for a sample pair involving different patients was below 10^−304^. From these results, we conclude that SMaSH is able to reliably identify patient matches even between different sequencing data types.

### Performance compared to similar software

**Fig. 2:**
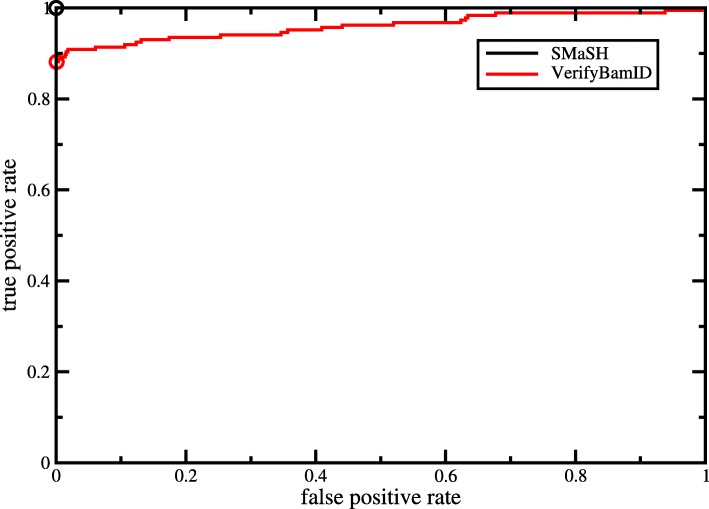
Receiver Operating Characteristic curves for the performance of SMaSH and VerifyBamID on a subset of data set 2 consisting of RNA-Seq and MethylCap-Seq libraries. Each curve shows the fraction of true positives as a function of the fraction of false positives. The black solid curve (which follows the axes as SMaSH is a perfect classifier on this data set) represents SMaSH and the red curve represents VerifyBamID. The circles indicate the performance at a *p*-value/IBD cutoff of 0.95

A subset of the second data set consisting of 31 RNA-Seq libraries and 9 MethylCap-Seq libraries was used for comparison with VerifyBamID [12], a tool with the same intent as SMaSH, which however relies on a separate SNP calling step ahead of the evaluation of sample identity. We elected to use a subset instead of the full data set due to the computational requirements of calling SNPs for VerifyBamID. We called SNPs on this data subset using samtools and bcftools [13]. To verify identity, we used VerifyBamID’s identity by descent (IBD) statistic. An IBD closer to 0 indicated the two samples were less related, while an IBD closer to 1 indicated the two samples were potentially from the same patient. Figure 2 shows the IBD of VerifyBamID compared against SMaSH’s classification and indicates that SMaSH was able to better identify samples originating from the same patient across data types.

### Performance on lower quality and low coverage data sets

**Fig. 3:**
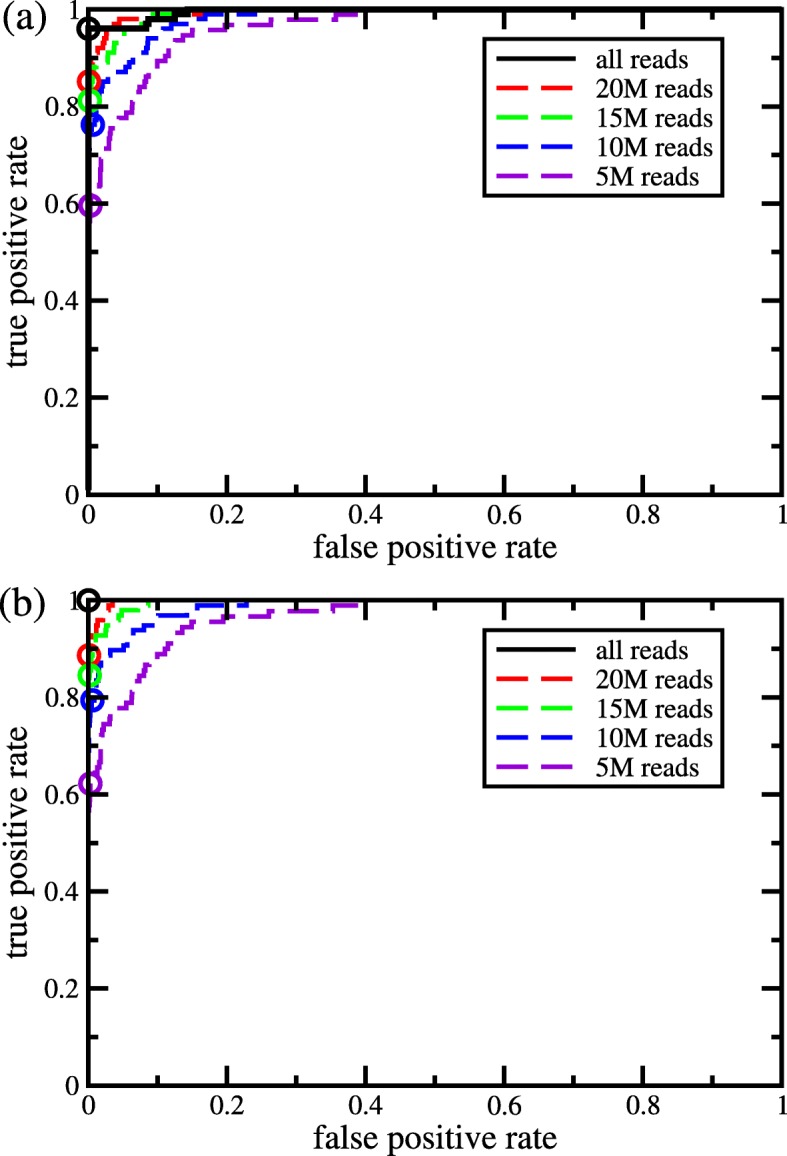
Receiver Operating Characteristic curves for the performance of SMaSH on a fairly low quality RNA-Seq data set. Each curve shows the fraction of true positives as a function of the fraction of false positives. The black solid curves correspond to the full data sets while the colored dashed curves correspond to different degrees of subsampling in order to illustrate how performance depends on read coverage. (a) shows data for all samples while (b) shows data after removal of all comparisons involving one sample that was later excluded from the study due to very low RNA quality. The circles indicate the performance at a *p*-value cutoff of 0.95

Next, we tested SMaSH on a whole transcriptome data set that due to its library preparation had lower than usual RNA quality. A few representative Perkin Elmer Labchip GX tracings reflecting the lower quality of these samples in the barely visible 18S and 28S ribosomal RNA peaks are available in Additional file 2. Quality control parameters determined using RNA-SeQC [14] for these samples are available in Additional file 3. The data set consisted of several technical replicates and tumor-normal pairs. We were initially blinded to all sample identities in this study; yet, as can be seen in Figure 3 we were able to nearly completely match samples originating from the same patient, despite the lower quality of the data. The few false negative sample pairs visible in Fig. 3a all turned out to include a single, problematic sample (sample RNA09). This sample was later rejected from further analysis because of failed QC (duplication rate of 0.68; see Additional file 3). Figure 3b shows the ROC curves excluding the rejected sample.

**Fig. 4:**
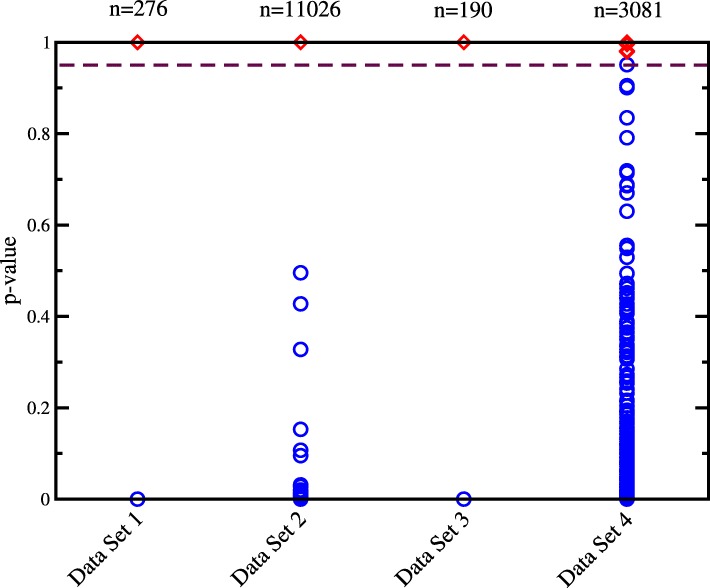
*p*-value distributions for all four data sets. Each symbol corresponds to the comparison of one pair of samples in the respective data sets and its height represents the calculated probability that the two samples are derived from the same individual. Red diamonds indicate sample pairs from the same individual while blue circles indicate sample pairs from different individuals. For data set 4 data after exclusion of the failed quality control sample RNA09 is shown. The dashed line corresponds to the chosen threshold of 0.95 that discriminates pairings involving the same individual from pairings not involving the same individual in all four data sets

In order to evaluate the effect of coverage, we also subsampled each sample from approximately 35 million reads per sample, to 20 million, 15 million, 10 million, and 5 million reads per sample. As seen in Fig. 3, as the data quality and coverage decrease, the ability of SMaSH to classify sample pairs also decreases. However, even with 5 million reads per sample the area under the curve (AUC) is still a respectable 0.967 or 0.966 with and without removal of the one low quality sample, respectively. It is important to point out that for the downsampled samples SMaSH reported “NOTEST” instead of a probability if there is no SNP with any coverage in both samples of a pair and these pairs were excluded from the evaluation. This affected 3 and 64 of the 3160 total pairs of samples in the 10 million read data set and the 5 million read data set, respectively. The lower coverage samples also allowed us to identify a *p*-value cutoff that corresponds to the upper left hand corner of the ROC curve (i.e., the point where false negatives start to become noticeable), and we found that a cutoff of *p*=0.95, i.e., at a 0.05 probability that the two samples are from different individuals, is optimal. As demonstrated in Fig. 4, this threshold (like many other possible choices) leads to perfect classification in the first three data sets as well.

### Performance on data sets with familial relationships

Finally, we applied SMaSH to samples sequenced from family members in order to test how well it performed when samples had a greater degree of genetic similarity. Exome data sets from a pair of siblings [15] and RNA-Seq data sets [16] from Family 1 (a mother, father, and their child) and Family 2 (a mother, father, and their two children) were tested. SMaSH calculated a probability of 7.8·10^−201^ that the pair of Exome samples were from the same individual and thus was clearly able to identify the two siblings as different individuals. The probabilities for the family RNA-Seq data are shown in Table 1. In Family 1, the null hypothesis of the samples being from the same patients was rejected at a 0.95 level for all sample pairs, even though the probability of the mother and the child being the same individual was reported as 0.16 and the probability of the father and the child being the same individual was reported as 0.80. In Family 2, the probability of the two children’s samples to have come from the same individual was calculated as 0.9999995 while the probability of the father and one of the children to have come from the same individual was calculated as 0.96. The other sample pairs were rejected at the 0.95 level. Given these findings, we do not recommend using SMaSH to identify sample pairs when multiple family members are suspected to be represented within the same dataset.

**Table 1:** Calculated probabilities that samples from members of two families come from the same individual

	Mother 1	Child 1	Father 2	Mother 2	Child 2	Sibling 2
Father 1	4 · 10^− 46^	0.80	2 · 10^− 151^	7 · 10^− 174^	1 · 10^− 161^	3 · 10^− 177^
Mother 1		0.16	2 · 10^− 171^	3 · 10^− 240^	8 · 10^− 237^	1 · 10^− 269^
Child 1			3 · 10^− 171^	6 · 10^− 175^	3 · 10^− 182^	4 · 10^− 198^
Father 2				1 · 10^− 78^	0.96	0.32
Mother 2					0.75	0.16
Child 2						0.9999995

## Discussion

We have shown that a Bayesian framework applied to read counts at a carefully selected set of about 6000 human SNPs is able to determine, with high reliability, if two NGS samples stem from the same individual or not, even if the data sets come from completely different sequencing types such as RNA-seq, Exome analysis, and MethylCap-seq. The key to the approach is that the evidence from many potentially weakly covered genomic locations is aggregated into a single *p*-value for the hypothesis that the two samples stem from the same individual.

We found that SMaSH performed perfectly for high quality samples not involving samples with genealogical relationships. However, as sample quality or sequence coverage decrease, false classifications occur. We noted that even in these challenging situations, the approach provides a ranking of sample relatedness, which may still be enough to detect possible sample swaps. Most likely such low quality samples would anyway have to be rejected, not because SMaSH fails to verify their identities, but because their quality is not sufficient for whatever downstream analysis the sample has been sequenced for.

Genealogical relationships between samples have a tendency to lead to false positives. While it is in principle possible to extend the framework to not only test for identity of individuals, but also allow parent-children relationships as alternative hypotheses, we found that such extension creates more false classifications on data sets without genealogical relationships due to increased permissiveness. We therefore did not further pursue this issue and currently recommend not to apply the tool to data sets with genealogical relationships, or at least, if doing so, not to count detected putative identities between related samples as sample swaps. However, even in the presence of genealogical relationships, the *absence* of a detected identity between samples that should correspond to the same individual *is* an indication of a sample swap.

One may ask if our approach is sensitive to race and/or ethnicity of the samples. Our list of SNPs is derived from the 1000 genomes project [17], which includes individuals from a broad set of races and ethnicities. Also, it has been estimated that only 15% of all human SNPs are population-specific [18], implying that most SNPs are shared across populations. Thus, we would not expect that our set of SNPs works preferentially for a particular race or ethnicit; however, we were not able to explicitly test this assertion given that data sets 1 and 3 solely stem from Caucasian patients and we were blinded to race and ethnicity information in data sets 2 and 4.

The advantage of our approach using a fixed set of SNPs is that for the purpose of identifying sample relationships within a study or across studies it is not necessary to access alignment files for every test but it is enough to count the number of reads supporting wild type and alternative alleles at each of the about 6000 locations for any future comparisons. In fact, it is most efficient to precompute the three quantities *Q*(*m*,*n*|*h*) for each SNP (see Eq. (3) in the “Methods” section), and, store these quantities in a database as a “fingerprint” of the sample for future comparisons. Furthermore, by using this fixed set of SNPs, SMaSH is able to work directly with aligned files and does not require variants to be called in a separate step, allowing it to be used earlier in a quality control and data processing pipeline.

While using a standardized set of SNPs can be advantageous for the reasons discussed above and while we were careful in constructing our set of SNPs to maximize the applicability of SMaSH to many different data types, the experimental design may require a different set of SNPs to be used. For example, if all data in a study comes from whole genome sequencing, expanding the set of SNPs beyond exon regions should improve the performance of the method, albeit at added computational cost. Since SMaSH reads the list of SNPs from a file in vcf format, it is easy for a user to provide a different or larger set of SNPs. However, increasing the set of SNPs may not be justifiable given the near perfect performance of our approach on multiple different data types using the selected set of about 6000 exonic SNPs).

One downside of our approach is that it currently cannot be applied to bisulfite converted data types [19]. Since in bisulfite conversion unmethylated cytosines are converted to uracils (which are read as thymines), some apparent alternative alleles are really a consequence of changes in methylation and/or cannot be detected at all. Thus, bisulfite conversion must be explicitly taken into account in the statistical model. Another avenue of future improvements is the development of a database of fingerprints of standard cell lines to enable the routine verification of cell line identities

## Conclusions

In summary, we have developed our tool SMaSH that is able to computationally aggregate SNP information to evaluate if two NGS samples are derived from the same individual or not. We have tested our tool on several unique data sets, established excellent performance on high quality data sets, and characterized how performance decreases for low quality, low coverage data sets. Most importantly, we have shown that our tool is able to detect matches between different sequencing data types. With this ability, our tool is ready to improve data integrity in modern multi-omics studies by verifying sample identity and reducing the impact of inadvertent sample swaps.

## Methods

### SNP selection

SMaSH uses a standard set of SNPs in order to test whether or not two NGS samples were generated from the same individual. We derived this standard set by first downloading all annotated SNPs from the 1000 genomes database [17] and selecting all SNPs *s* that had an allele frequency *q*_*s*_ in the range 0.1<*q*_*s*_<0.9 located throughout the entire genome. To decrease computation time and simultaneously increase the chance of finding SNPs occurring in multiple data types, such as Exome and RNA-Seq data sets, we then intersected these SNPs with the Illumina TruSeq Exome Enrichment Kit’s (TruSeq Exome Enrichment Kit Data Sheet, Illumina, San Diego, CA) targeted regions as well as the Agilent SureSelect (SureSelect Human All Exon V5 data sheet, Agilent Technologies, Santa Clara, CA) targeted regions. These regions are more likely to have coverage for SNP calling across all discussed data types: whole genome sequencing, RNA-seq, Exome-seq, and MethylCap-seq. To account for linkage equilibrium, we required each SNP to be at least 100kb away from any other SNPs in the list. In cases where SNP were closer than this minimum distance, we chose the SNP with the allele frequency closest to 1/2 in order to maximize the information content contributed by the SNP. This resulted in 6059 SNPs to be tested, which are listed in Additional file 4.

### Algorithm

For each sample being tested, SMaSH iterates over every SNP in the 6059 SNPs dataset. For each SNP *s*, SMaSH uses pysam [13] to determine the total number of reads covering the SNP, the number *m*_*s*_ of reads that match the reference nucleotide, and the number *n*_*s*_ of reads that do not match the reference nucleotide.

After gathering read information, SMaSH employs a Bayesian model to calculate the probability that two samples are derived from the same individual. To this end, we introduce a variable *x* that takes the value I if the two samples are from the same individual and the value D if the two samples are from different individuals, with associated priors *π*_*I*_ and *π*_*D*_=1−*π*_*I*_, respectively. For the prior value we choose *π*_*I*_=0.01 but we noted that the specific choice of this value does not affect the results significantly.

We also introduce the apparent alternate allele frequency *f*_*s*_ ∈[0,1] for each SNP in a sample and the genotype *h*_*s*_ of SNP *s* in a sample. The latter can have the values WW (homozygous wildtype), AA (homozygous alternate), and WA (heterozygous). The probability to observe read counts *m*_*s*_ and *n*_*s*_ from one sample and $m_{s}^{\prime }$ and $n_{s}^{\prime }$ from the other sample with the hidden variables taking values *f*_*s*_, $f_{s}^{\prime }$, *h*_*s*_, $h_{s}^{\prime }$, and *x*, respectively, is then given by

$$\begin{array}{@{}rcl@{}} \lefteqn{\Pr\{m_{s},n_{s},m_{s}^{\prime},n_{s}^{\prime},f_{s},f_{s}^{\prime},h_{s},h_{s}^{\prime},x\}=}\hspace*{1mm}&&\\ \quad&=&\pi_{x}\prod_{s} P(m_{s},n_{s}|f_{s})P(f_{s}|h_{s})\times\\ &&\hspace*{8mm} \times P(m_{s}^{\prime},n_{s}^{\prime}|f_{s}^{\prime})P(f_{s}^{\prime}|h_{s}^{\prime})P(h_{s},h_{s}^{\prime}|x). \end{array} $$

We model sequencing itself as a binomial process, i.e., we use

1 $$ P(m,n|f)={n+m \choose n}f^{n}(1-f)^{m}.  $$

For the apparent alternate allele frequencies *f* we expect values close to zero (but not quite zero due to sequencing errors) for *h*=WW, values close to one for *h*=AA, and values around 1/2 for *h*=WA. To model these expectations, we choose a beta distribution

2 $$ P(f|h)=\frac{f^{\alpha_{h}-1}(1-f)^{\beta_{h}-1}}{B(\alpha_{h},\beta_{h})}  $$

with *α*_WW_=*β*_AA_=1, *β*_WW_=*α*_AA_=30, and *α*_WA_=*β*_WA_=2, where *B*(*α*,*β*) is the beta function. Since we will apply a Bayesian approach, the relevant combination of these two probability distributions is

3 $$\begin{array}{@{}rcl@{}} Q(m,n|h)&=&\int_{0}^{1} P(m,n|f)P(f|h)\mathrm{d}f\\ &=&{n+m \choose n} \frac{B(\alpha_{h} + n, \beta_{h} + m)}{B(\alpha_{h}, \beta_{h})}. \end{array} $$

We then need to calculate

4 $$\begin{array}{@{}rcl@{}} \lefteqn{\Pr\{x|m_{s},n_{s},m_{s}^{\prime},n_{s}^{\prime}\}\sim}&&\\ &&\pi_{x}\prod_{s}\sum_{h_{s}}\sum_{h_{s}^{\prime}} Q(m_{s},n_{s}|h_{s})Q(m_{s}^{\prime},n_{s}^{\prime}|h_{s}^{\prime})\times\\ &&\hspace*{45mm}\times P(h_{s},h_{s}^{\prime}|x). \end{array} $$

This can be most conveniently done by arranging the integrals *Q*(*m*,*n*|*h*) for each SNP *s* into a 3x3 matrix $\widehat {Q}_{s}$ with matrix elements

5 $$ (\widehat{Q}_{s})_{h,h^{\prime}} = Q(m_{s},n_{s}|h)Q(m_{s}^{\prime},n_{s}^{\prime}|h^{\prime}).  $$

Similarly, the term $P(h_{s},h_{s}^{\prime }|x)$ in Eq. (4) can be written as one 3x3 matrix $\widehat {I}_{s}$ for *x*=I (same individual) and another 3x3 matrix $\widehat {D}_{s}$ for *x*=D (different individuals). These are expressed in terms of the population allele frequency *q*_*s*_ of the current SNP being tested as

$$\begin{array}{@{}rcl@{}} \widehat{I}_{s}&=&\left(\begin{array}{ccc} (1-q_{s})^{2} & 0 & 0 \\ 0 & 2q_{s}(1-q_{s}) & 0 \\ 0 & 0 & q_{s}^{2} \\ \end{array}\right)\quad\text{and}\\ \widehat{D}_{s}&=&\left(\!\begin{array}{ccc} (1-q_{s})^{4} & 2q_{s}(1-q_{s})^{3} & q_{s}^{2}(1-q_{s})^{2} \\ 2q_{s}(1-q_{s})^{3} & 4q_{s}^{2}(1-q_{s})^{2} & 2q_{s}^{3}(1-q_{s}) \\ q_{s}^{2}(1-q_{s})^{2} & 2q_{s}^{3}(1-q_{s}) & q_{s}^{4} \\ \end{array}\!\right)\!. \end{array} $$

Finally we obtain the probability that the two samples are from the same individual as

6 $$\begin{array}{@{}rcl@{}} \lefteqn{\Pr\{x=I|m_{s},n_{s},m_{s}^{\prime},n_{s}^{\prime}\}=}\hspace*{10mm}&&\\ \quad&&\frac{\pi_{I}\prod_{s}\text{Tr}(\widehat{Q}_{s}\widehat{I}_{s})} {\pi_{D}\prod_{s}\text{Tr}(\widehat{Q}_{s}\widehat{D}_{s})+\pi_{I}\prod_{s}\text{Tr}(\widehat{Q}_{s}\widehat{I}_{s})}, \end{array} $$

where Tr is the trace over the 3x3 matrices.

### Receiver operating characteristic analysis

The tradeoff between sensitivity and specificity of our algorithm was quantified using Receiver Operating Characteristic (ROC) analysis. To this end, we precalculated the *Q*_*s*,*i*_ defined in Eq. (3) for every SNP *s* in each of the *N* samples *i* of a data set. This allowed us to efficiently calculate the probability *p*_*i*,*j*_ that samples *i* and *j* derive from the same individual using Eq. (6) for each sample pair (*i*,*j*) with *i*<*j*. We also used the experimental design of the respective data sets to determine the reference set ${\mathcal {T}}\,=\,(i,j)| \text {{i} and {j} derive from the same individual}$

of true positives. We then sorted the *p*_*i*,*j*_ and calculated for each cutoff value *p* in this list the true positive rate

7 $$ TPR=\frac{|\{(i,j)\in {\mathcal{T}}|p_{i,j}\ge p\}|}{|{\mathcal{T}}|}  $$

and the false positive rate

8 $$ FPR=\frac{\{(i,j)\not\in {\mathcal{T}}|p_{i,j}\ge p\}|}{\frac{N(N-1)}{2}-|{\mathcal{T}}|}.  $$

The ROC curve is then obtained by plotting the true positive rate as a function of the false positive rate.

### Sample preparation and alignment

In order to thoroughly test our algorithm, we applied it to a variety of data sets and compared it against a similar tool, VerifyBamID [12]. Some of these were public data sets but others were generated in house as part of the normal operation of our sequencing facility. One data set (referred to as data set 3 in this manuscript) was obtained from a TCGA study on lung squamous cell carcinoma [20]. Metadata concerning this data set is available in Additional file 5. Exome data from a sibling pair [15] was downloaded from SRA under accession code ERX149719. Family RNA-Seq data [16] was obtained from the European Nucleotide Archive under accession numbers PRJEB5063 and PRJEB3030 (SAMEA1325278). The rest of this section describes the library preparation, sequencing, and alignment steps for the in house data sets.

Data set 1 and 4 libraries were generated using the stranded total transcriptome Illumina kit (TruSeq Stranded Total RNA LT with Ribo-Zero Gold; RS-122-2301). Total RNA was quantified using Invitrogen Qubit 2.0 Fluorometer (Life Technologies, Grand Island, NY) and 200 ng was used as input. Samples were required to have a RIN (RNA Integrity Number) >4 as assessed with Agilent 2100 Bioanalyzer RNA 6000 Nanochip (Agilent Technologies, Santa Clara, CA). Libraries with compatible barcodes were pooled and sequenced on Illumina HiSeq 2500 Sequencer using High Output Mode to achieve 35 to 40 million passed filtered paired-end 50bp reads per sample.

Data set 2 consisted of RNA-Seq and MethylCap-Seq libraries. RNA-Seq libraries were generated using the non-stranded Illumina kit (TruSeq RNA Sample Prep Kit; RS-122-2001). Total RNA was quantified using Invitrogen Qubit 2.0 Fluorometer (Life Technologies, Grand Island, NY) and 200 ng was used as input. Samples were required to have a RIN >7 as assessed with Agilent 2100 Bioanalyzer RNA 6000 Nanochip (Agilent Technologies, Santa Clara, CA). Libraries with compatible barcodes were pooled and sequenced on Illumina HiSeq 2500 Sequencer using High Output Mode to achieve 35 to 40 million passed filtered single-end 50bp reads per sample. MethylCap-Seq libraries were prepared by enriching methylated DNA fragments (150-200 bp) using the methyl binding domain (MBD) of human MeCP2 (Auto MethylCap Kit, Diagenode, Denville, NJ) on the Diagenode SX-8G IP-Star Automated System as described by the manufacturer’s protocol. The methylated DNA fragments were eluted with 1M NaCl. Illumina sequencing libraries were generated from the enriched methylated material as previously described [21]. Library materials were quantified by fluorometric measurement and quality of the samples was assessed by Agilent Bioanalyzer High Sensitivity DNA analysis prior to sequencing on the Illumina HiSeq 2500 flow cells. Images were captured from the sequencer and analyzed using the Real Time Analysis (RTA) software yielding 50 bp single-end sequenced reads.

RNA-Seq reads in all data sets were adapter trimmed using AdapterRemoval [22] and highly abundant species, such as human rRNA, were removed by aligning with Bowtie2 [23] and discarding aligned reads. The remaining reads were aligned using STAR [24] to the human genome version hg19. Methylation reads were collapsed to remove duplicated reads produced by polymerase chain reaction artifacts. Non-duplicate reads were aligned to hg19 using Bowtie allowing for two mismatches in a 32 bp seed while suppressing all reads that mapped to multiple locations in the genome.

## Supplementary information

**Additional file 1** This excel spreadsheet includes *p*-values for every pairwise comparison of two samples used in the evaluation of SMaSH.

**Additional file 2** The Labchip tracings in this supplementary figure document the sample quality in the lower quality RNAseq data set used in the evaluation of SMaSH.

**Additional file 3** This excel spreadsheet contains sample by sample quality control parameters of the lower quality RNASeq data set used in the evaluation of SMaSH.

**Additional file 4** This excel spreadsheet lists the 6059 SNPs used by SMaSH for identity assessment.

**Additional file 5** This excel spreadsheet lists metadata for the samples from the lung squamous cell carcinoma data set used in the evaluation of SMaSH.

## Data Availability

The source code of SMaSH implemented in python 2.7 is freely available for non-for-profit applications at http://github.com/rbundschuh/SMaSH.

## Abbreviations

DNA: DeoxyriboNucleic Acid
IBD: Identity By Descent
LGRC: Lung Genomics Research Consortium
NGS: Next Generation Sequencing
RIN: RNA Integrity Number
RNA: RiboNucleic Acid
ROC: Receiver Operating Characteristic
RTA: Real Time Analysis
SMaSH: Sample Matching using SNPs in Humans
SNP: Single Nucleotide Polymorphism
SRA: Short Read Archive
STAR: Spliced Transcripts Alignment to a Reference
STR: Short Tandem Repeat
TCGA: The Cancer Genome Atlas
UNC-LCCC: University of North Carolina Lineberger Comprehensive Cancer Center
