# Supplementary material for: SMaSH: Sample matching using SNPs in humans
Source: BMC Genomics. 2019 Dec 30;20(Suppl 12):1001. doi: 10.1186/s12864-019-6332-7 (PMC6936078; doi:10.1186/s12864-019-6332-7)
Supplement: Supplementary file 2 — Additional file 2 The Labchip tracings in this supplementary figure document the sample quality in the lower quality RNAseq data set used in the evaluation of SMaSH. [file 12864_2019_6332_MOESM2_ESM.pdf]

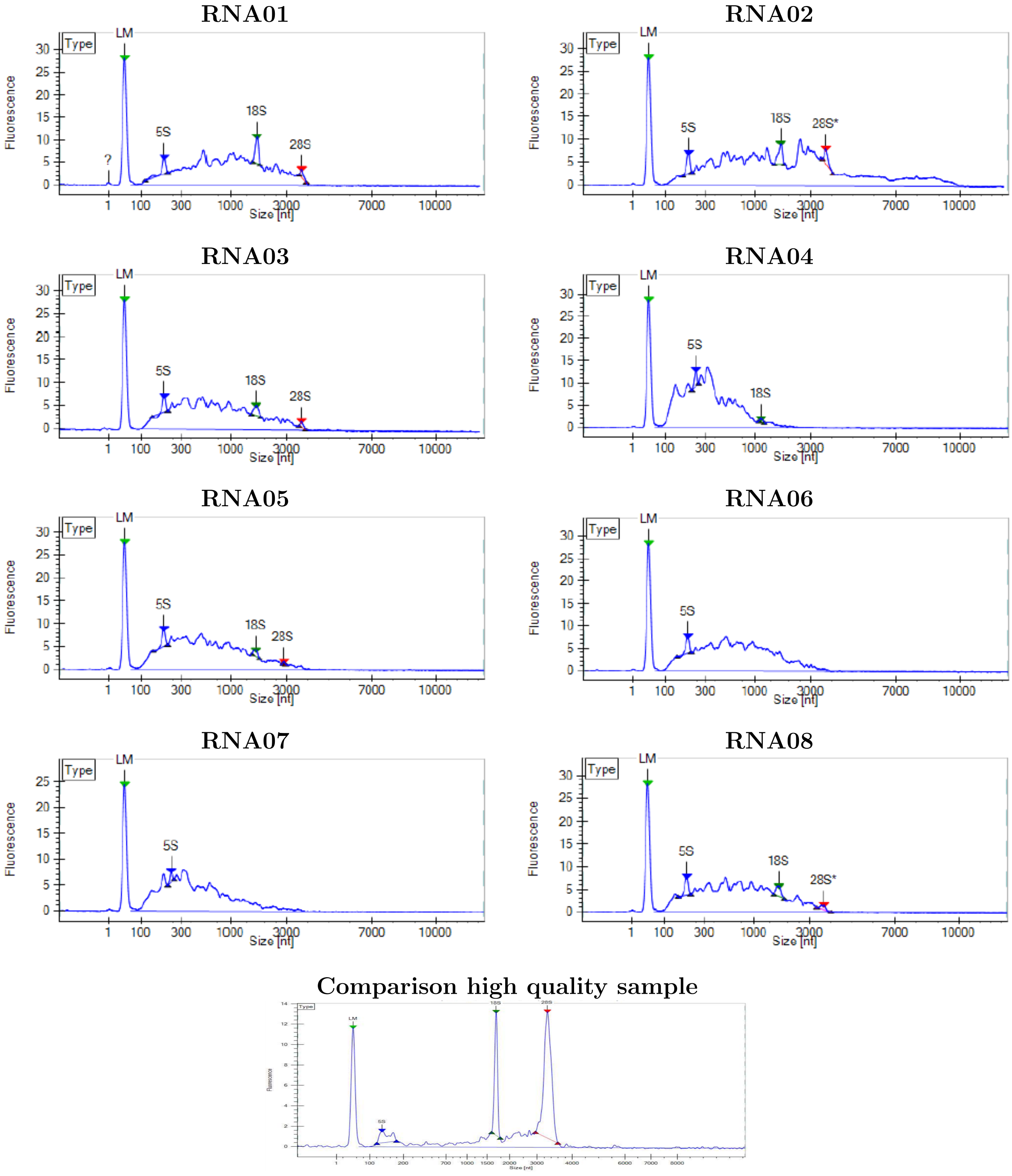

Supplementary Figure S1: Representative Perkin Elmer Labchip GX tracings of the first eight RNA samples of data set 4. The 18S and 28S peak are barely or not at all visible indicating the low RNA quality of these samples. As a comparison, a tracing from an unrelated, high quality sample is shown at the bottom.
